# Performance of the London Atlas, Willems, and a new quick method for dental age estimation in Chinese Uyghur children

**DOI:** 10.1186/s12903-022-02652-x

**Published:** 2022-12-21

**Authors:** Yueting Lin, Nuerbiyamu Maimaitiyiming, Meizhi Sui, Nuerbiya Abuduxiku, Jiang Tao

**Affiliations:** 1 Department of General Dentistry, Shanghai Ninth People’s Hospital, Shanghai Jiao Tong University School of Medicine; College of Stomatology, Shanghai Jiao Tong University; National Center for Stomatology; National Clinical Research Center for Oral Diseases; Shanghai Key Laboratory of Stomatology; Shanghai Research Institute of Stomatology, No. 500 Qu Xi Road, Shanghai, 200011 China; 2 Department of Cariology and Endodontics, The First Affiliated Hospital of Xinjiang Medical University (The Affiliated Stomatology Hospital of Xinjiang Medical University), Ürümqi, Xinjiang China; 3 Department of Stomatology, Kashgar Prefecture Second People’s Hospital, Kashgar, Xinjiang China; 4 Department of Stomatology, The First People′s Hospital of Kashgar Prefecture, No. 66 Yingbin Avenue, Kashgar, Xinjiang 844000 China

**Keywords:** Dental age estimation, Methodology, Chinese Uyghur, Children

## Abstract

**Background:**

Numerous dental age estimation methods have been devised and practised for decades. Among these, the London Atlas and Willems methods were two of the most frequently adopted, however dependent on atlantes or tables. A new estimation method less reliant on external measurement could be efficient and economical.

**Aim:**

This study aimed to evaluate the utility and applicability of the dental age estimation methods of London Atlas, Willems, and a new quick method that subtracts the number of developing teeth from the universal root mature age of 16 years in one of the lower quadrants reported in this work among Chinese Uyghur children.

**Methods:**

A comparative cross-sectional study was conducted. Subjects enrolled in the study were screened according to preset inclusion and exclusion criteria. The observer then obtained the dental age from the subjects’ panoramic radiographs based on the estimated rules of the London Atlas, Willems, and a new quick method. Paired *t*-test was used to compare the accuracy and precision of the above three estimation methods. Independent-sample t-test was used to find the difference between gender.

**Results:**

Totally, 831 radiographs entered the analyses of this study. Among the three methods evaluated, the Willems method, in particular, showed a distinct underestimated tendency. The mean error of the dental age predicted by the London Atlas, the Willems method, and the quick method was 0.06 ± 1.13 years, 0.44 ± 1.14 years, and 0.30 ± 0.63 years, respectively. The mean absolute error was 0.86 ± 0.75 years according to the London Atlas, 1.17 ± 0.89 years under the Willems method, and 0.70 ± 0.54 years under our quick method. No significant difference was found between the chronological age and dental age using the London Atlas, generally for the 10 to 15 years group (*p* > 0.05), but our quick method for the 15–16 years children (*p* < 0.05) and Willems method (*p* < 0.001).

**Conclusion:**

The London Atlas outperformed the Willems method with better accuracy and precision among 10–15 years Chinese Uyghur children. Our new quick method may be comparable to the London Atlas for children aged 10–14 and potentially become a more straightforward dental age prediction instrument.

**Supplementary Information:**

The online version contains supplementary material available at 10.1186/s12903-022-02652-x.

## Introduction

Determining one’s chronological age (CA) is crucial in the cases of identifying the identity of a corpse in forensic sciences, a criminal or suspicion in judicial practice, an orphan in adoption procedures [1], a patient in orthodontic treatment [2], et al. Some studies obtain the chronological age by analyzing one’s skeletal maturation. Nevertheless, dentition shows greater environmental stability than the skeleton during growth [3], making dentition an excellent indicator for chronological age estimation, known as dental age (DA) estimation.

Dental age estimation is the methodology to predict the chronological age by identifying the characteristics and features of the developmental teeth (e.g., the root resorption of the deciduous tooth [4], the mineralization or eruption of the permanent teeth [5]) using a radiograph.

As of the 1960s, numerous dental age estimation methods were emerging. And their way of working was various. The Demirjian [6, 7], Willems [8], Nolla [9], and Häävikko [10] methods quantified the calcification progression of teeth with different stage-classification and scoring systems. The London Atlas determined age by matching a panoramic radiograph to a sequential tooth development reference atlas [11]. The Kvaal method measured the length and width of the pulp/root/tooth and related them to age with regression models [12]. The Cameriere method calculated DA with a formula correlated to the pulp/tooth area ratio [13] or open apices in teeth [14]. The former methods estimated age by developing teeth and were mainly applied to children. And the last two, which analyzed the secondary dentin indirectly, were primarily used in adults.

Although age-estimation methods were manifold, they still need practising and testing in different populations for the ethnic and regional differences in the development of teeth. During the past decade, some investigators used to explore and concluded that the Willems method was a more appropriate method than the Demirjian, Nolla, and Häävikko method in children and adolescents from India [15], Malaysia [16, 17], Brazilia [18], Bangladesh and British Caucasian area [5], etc. And the London Atlas could match the Willems method [3, 19].

Previously, we had ever applied some of the methods above to the southern and eastern Chinese populations and finally filtered out the corresponding applicable methods [20, 21]. And in this study, we collected the panoramic radiographs of northwestern Chinese Xinjiang Uighur children to investigate the utility and suitability of the London Atlas, Willems method, and a new quick method for dental age estimation. Further, to complete the dental age prediction data gap for the Chinese Uighur youth ethnic group.

## Materials and methods

### Sample collection

This comparative cross-sectional study adopts the simple random sampling method. The case report form was used in the data collection. Digital orthopantomograms (OPGs) in our study were from patients with the essential need for oral disease diagnosis and treatment. And the Uyghur samples recorded were mainly from Urumchi and Kaxqar in northwestern China. Cases aged 10.00 to 16.99 were included. The exclusion criteria involve bilateral teeth loss, images with blur or distortion, congenital anomalies, orthodontic therapy history, and dental trauma that may affect the maturity of teeth.

### Dental age calculation

The London Atlas comprises a series of schematic images of dentition development for every age. Investigators assessed dental age by referring to the documented atlas. The corresponding age of the best-matched panoramic radiographs of the patients being examined to the reference image was considered DA [11]. To match the image as close as possible and reduce the indecisive dilemma when a tooth appeared between stages, we employed the in-between estimates in the London Atlas.

Willens method is a revised version with adjusted scores of Demirjian’s method, which categorized the tooth development into eight calcification stages from calcified points formation to apical foramen closure. Investigators used this method by assigning the left mandibular tooth (the 3rd molar excepted) to a stage of A-H according to the morphology and features of the developing teeth. Each stage was then converted into a score based on the previously published gender-specific tables. And the scores of all seven teeth were summed up as the final estimated DA [8].

Our quick method could be interpreted as a straightforward subtraction operation. In light of the pattern that 16 years is the age generally when the dentition excluded from the 3rd molar completely developed, we use 16 as the minuend and the number of developing teeth with incomplete roots in one of the lower quadrants as the subtrahend, then take the arithmetic result that subtracting the subtrahend from the minuend as the predicted DA. The principle behind this method may be that the teeth mature by year in one of the lower quadrants, roughly from 9 to 15 years.

The performance of the three methods was undertaken by one professional investigator. When assessing, the investigator zoomed in on the pictures to observe the details of the periodontal ligament and apical end. Ten months later, the same investigator conducted the intra-observer reliability using 5% randomly selected samples. Another well-trained investigator with 5% identical samples carried out the inter-observer reliability.

### Statistical analyses

The statistical analysis was conducted using the Statistical Package for Software Science (IBM SPSS Version 25.0). By subtracting the birth date from the OPGs date taken, we calculated chronological age (CA) and corrected all ages to two decimal places. The seven age brackets with gender divided for all the panoramic samples were used. To display the direction of overestimation and underestimation (i.e., a positive value indicates an underestimate), the mean age difference, also described as the mean error (ME), was computed. Additionally, mean absolute error (MAE) between CA and DA was bias independently employed to gauge the accuracy and precision of the three methods.

The paired *t*-test was used to examine the individual accuracy of the London Atlas, Willems methods, and the quick method, with a standard deviation (SD) and a 95% confidence interval (CI), by comparing the significant difference in estimated age between CA and DA among each age group. Besides, the independent-sample *t*-test was adopted to investigate the estimated difference in the three methods between boys and girls. All the tests were two-sided, and the level of statistical difference to be considered significant was set at 0.05. The intraclass correlation coefficient (ICC) test was employed for checking the intra- and inter-examiner agreements.

## Results

**Fig. 1:**
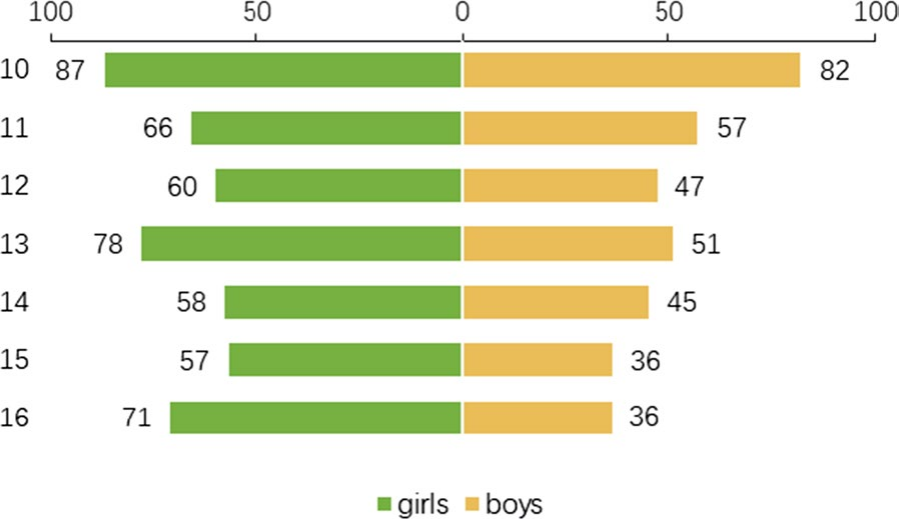
Distribution of gender in different age

Of all the 982 OPGs collected, 831 cases were qualified for age estimation. Girls account for 57.4%, and boys for 42.6%. Girls and boys in every age cohort were balanced generally (Fig. 1). 10 years group was the largest, while 15 years group was the smallest. The youngest and oldest subject was 10.00 and 16.99 years, respectively. All three methods yielded a respective ICC score, indicating good reliability (Additional file 1: Table S1).

**Fig. 2:**
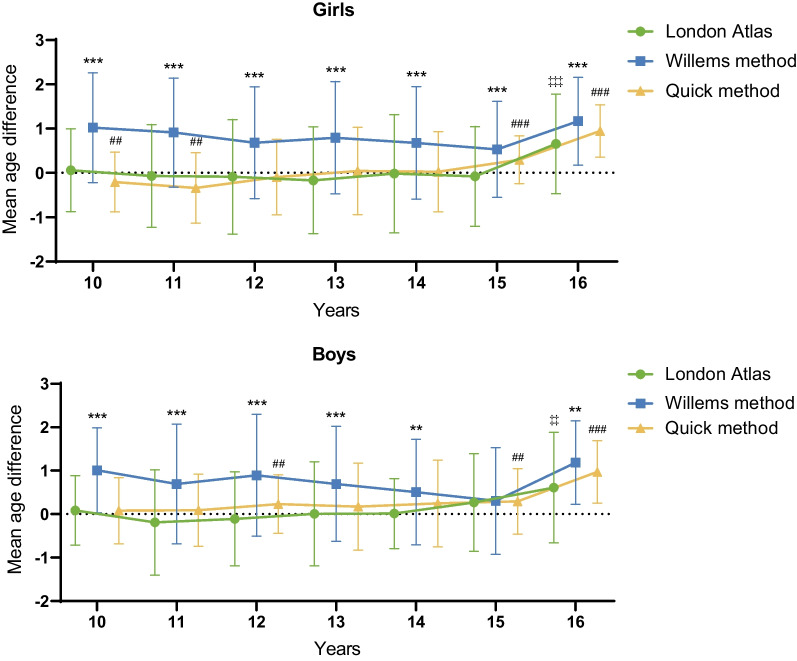
Mean age difference among different ages, genders, and methods (*, *p* < 0.05; **, *p* < 0.01; ***, *p* < 0.001. ‡, *p* < 0.05; ‡‡, *p* < 0.01; ‡‡‡, *p* < 0.001. #, *p* < 0.05; ##, *p* < 0.01; ###, *p* < 0.001)

When exploring the age estimation accuracy of three tested methods within each group, the 16 years group showed a significant difference in all London Atlas, Willems methods, and the quick method (*p* < 0.01). And the 15 years group of boys exceptionally displayed no statistical difference in Willems’s method (*p* > 0.05). As for the quick method, it generally showed acceptable estimations in 10–14 years (*p* > 0.05), though boys of 12 years and girls of 10 or 11 years may exhibit less accuracy (*p* < 0.05) (Additional file 1: Table S2 and Fig. 2).

Overall, the dental age assessed by the London Atlas revealed no statistically significant difference (*p* = 0.148), which denoted no difference between estimated DA and CA in the London Atlas. However, the estimated age by the Willems method and our quick method manifested differences from CA (*p* < 0.001). Specifically, the mean difference of the London Atlas in total was 0.06 ± 1.13 years, and the mean absolute error was found to be 0.86 ± 0.75 years. Whereas the mean difference of the Willems method was 0.44 ± 1.14 years, and the mean absolute error was 1.17 ± 0.89 years, which indicated less accuracy and precision than the London Atlas’ (p < 0.001). In addition, our quick estimated method showed a mean difference of 0.30 ± 0.63 years and a mean absolute error of 0.70 ± 0.54 years, which suggested that the accuracy and precision of it may be comparable to that of the London Atlas.

**Fig. 3:**
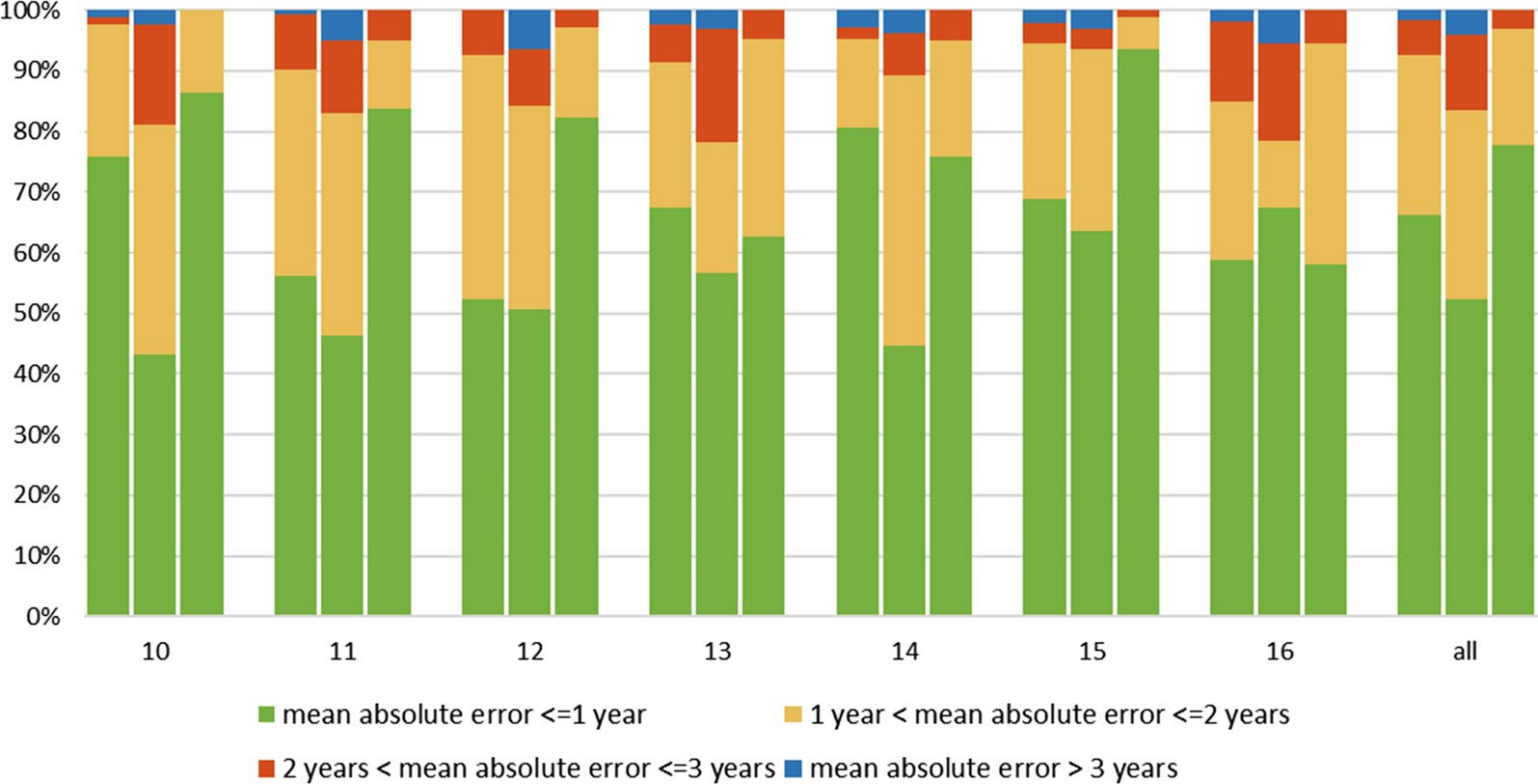
Comparison of the accuracy of predicted dental age by year (Left: the London Atlas; Middle: the Willems method; Right: the quick method)

On the other hand, the error of the estimated age by-year interval exhibited that the London Atlas was more applicable than the Willems method. It is, meanwhile, demonstrated that the quick method outperformed the London Atlas and the Willems method from a different perspective. Regarding the London Atlas, the proportion of subjects whose age error between CA and DA that less than one year was 66.2%, and Willems 52.2%, while 77.6% better using the quick method. And the age error ranges from 1 to 2 years was 26.5% in the use of London Atlas, 31.2% in the Willems method, and 19.1% in the quick method. Conversely, compared to the results within one year, when the estimated age was more than two years, the percentage of age error using the Willems method exceeded the London Atlas and the quick method with 16.6%, 7.4%, and 3.2%, respectively (Additional file 1: Table S3 and Fig. 3).

**Fig. 4:**
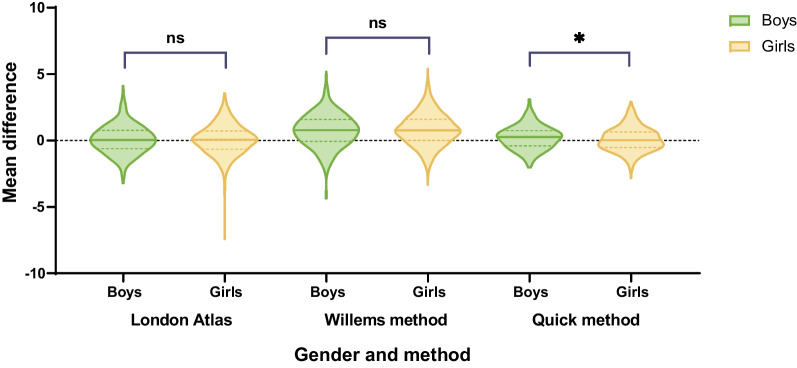
Violin plot of the MAE between genders using London Atlas, Willems method, and quick method

Concerning the role and factor of gender in the age prediction, no significant difference was found (*p* > 0.05) in both the London Atlas and Willems methods but in the quick method (*p* < 0.05). Moreover, it was shown that the distribution of the medians of the mean age difference was closer to zero and more concentrated in the London Atlas and the quick method, contrary to the Willems method (Fig. 4), which was scattered and presented a more underestimated tendency.

Finally, since the original age parameter of 16 minus CA and the number of immature teeth (i.e., the error between CA and DA) should be as close to zero as possible, the age parameter in the current study could be adjusted and corrected to 16.25 years for boys and 16.09 years for girls averagely or more specific for every group (Additional file 1: Table S4).

## Discussion

People of different ethnicity often possess heterogeneous tooth growth patterns, while individuals of the same ethnic groups usually have similar tooth development laws [22]. Hence, age estimation methods were recommended for testing and practice in diverse populations. Previously, lots of studies surveyed the dental age among different samples using different methods. Here, In the current study, we probed and exhibited the validity and applicability of three dental age estimation methods in various ways.

For age prediction methods, accuracy is a primary criterion. Since the accuracy of a dental age estimation method refers to the extent of the value measured close to the chronological age [23], the dental age predicted by London Atlas was feasible with no significant statistic difference between CA and DA (*p* > 0.05) except for the 16 years group (*p* < 0.001). And the quick method in 10–14 years was acceptable and of competitiveness with 1 year mean error. By comparison, the age estimated by the Willems method showed a slight tolerated error within 1.5 years, which yielded a significant difference (*p* < 0.05) from CA. The results of the Willems method were more or less similar to previous research in the Eastern Turkish and Tunisian populations [3, 24]. In contrast, the Croatian and Sri Lankan population-specific studies reported a lower error rate using the Willems method [25, 26]. In addition, the dental age estimates also show that the London Atlas was more accurate than Willems’ method (*p* < 0.001), which is in accordance with literature that sampled children with molar-incisor hypomineralization [27], though Alaettin Koç and Bianca Gelbrich’s early study [3, 19] displayed no statistical precision difference between those two methods (*p* > 0.05).

Another accuracy indicator of a dental age estimating method is dental age with a mean prediction error of less than or equal to one year. A more than one-year bias may limit the practicability of an age-estimated method [28]. In our Uygur population, the margin of error within one year using the London Atlas, the Willems method, and the quick method was lower than the sample of Brazilians, Kenyans and Hispanics [29–31] but higher than studies on a Spanish and Somali [32, 33], etc. The estimation error of more than two years in the London Atlas was 7.4%, which was similar to a study on German samples [19]. However, the prediction error that more than two years of the Willems method was 16.6%, which exceeded some other studies [19, 24]. Although several studies have found that females are more likely to be overestimated than males for their faster growth pace of teeth and body [3, 15], our study showed that no gender differences were found in the London Atlas and Willems method. It may own to the internal calibration of these computed methods themselves. And unsurprisingly, there was a statistical difference calculated between genders in our quick method, which informed us of the further improvement of it and the information on how to make the adaptive adjustment based on this study when conducting future predictions.

Some researchers also claimed that the younger the subjects are, the more accurate the result would be [34, 35]. While in the current study, the 16-year group showed a more significant bias than the younger group, the association between accuracy and chronological age remained unclear.

The factors that affected the accuracy and precision of the outcome could be intricate. Inherently, it is the inevitable different growth patterns of the individual matter. According to previous studies, genetic elements account for 90% of tooth development, with environmental factors having less influence [22]. Given the unique climate and lifestyle of the Xinjiang Uygur region, we considered environmental factors such as nutrition, customs, and sunlight exposure [36, 37] et al. could be factors that impact the dental development process in the Xinjiang Uygur population. Nevertheless, we did not record the information on the potential factors of each subject, thus limiting the causal analysis in further exploration.

In terms of the limitation of the quick methods, one shortcoming is that only individuals aged between 10–15 years could be calculated. Dental age estimation for people younger or older outside the age range still requires additional indicators to expand the applicability (e.g., the eruption sequence of permanent teeth for the lower than 9 years group and the characteristics of the third molar for the higher than 16 years group). Secondly, the shortcoming of the current method may exist in the precision provided. Hence more classification or refinement on the year interval may increase the hit of the actual age.

The objective of the present article was to compare and determine the validity of three dental age estimation methods in the Chinese Uygur population. In general, the London Atlas and the quick method were more applicable. However, when using the London Atlas, we may need to memorize a series of different pictures or do a reference with the help of outside instruments, while the quick method could not have to.

In this study, we included samples of homogeneous Uygur backgrounds, reported a quick dental age prediction method, and tested two widely used methods comparatively. Further research may focus on the factors that affect Uygur tooth maturation and human development. And our quick method may need some elaboration on its precision, or the other age prediction method may be applied or invented in the Uygur ethnic population so that the least variable method could be chosen.

## Conclusion

In the present study, we reveal information concerning the age prediction of Xinjiang Uygur youth using the London Atlas, Willems method, and a quick method. Overall, The London Atlas was more practical than the Willems method for age estimation in 10–15 years Uygur children. And the quick method was comparable to the London Atlas with acceptable accuracy and the advantage of being more time-saving and convenient.

## Supplementary Information

**Additional file 1. Table S1–S4. Table S1. **The intraclass correlation coefficient (ICC) test of the three methods.** Table S2. **Analysis of ME and MAE between CA and DA using the three methods (years).** Table S3. **Frequency of dental age estimation accuracy of the three methods in this study by year.** Table S4. **Age parameter correction for boys and girls.

## Data Availability

All data generated or analyzed during this study are included in this published article and its supplementary information files.

## Abbreviations

CA: Chronological age
DA: Dental age
ME: Mean error
MAE: Mean absolute error
SD: Standard deviation
CI: Confidence interval
ICC: The intraclass correlation coefficient test
OPGs: Orthopantomograms

## References

[1] Vila-Blanco N, Carreira MJ, Varas-Quintana P, Balsa-Castro C, Tomas I (2020). Deep neural networks for chronological age estimation from OPG images. IEEE Trans Med Imaging.

[2] Marroquin Penaloza TY, Karkhanis S, Kvaal SI, Vasudavan S, Castelblanco E, Kruger E, Tennant M (2017). Orthodontic treatment: real risk for dental age estimation in adults?. J Forensic Sci.

[3] Koc A, Ozlek E, Oner Talmac AG (2021). Accuracy of the London atlas, Willems, and Nolla methods for dental age estimation: a cross-sectional study on Eastern Turkish children. Clin Oral Investig.

[4] Heboyan A, Avetisyan A, Karobari MI, Marya A, Khurshid Z, Rokaya D, Zafar MS, Fernandes GVO (2022). Tooth root resorption: a review. Sci Prog.

[5] Maber M, Liversidge HM, Hector MP (2006). Accuracy of age estimation of radiographic methods using developing teeth. Forensic Sci Int.

[6] Demirjian A, Goldstein H (1976). New systems for dental maturity based on seven and four teeth. Ann Hum Biol.

[7] Demirjian A, Goldstein H, Tanner JM (1973). A new system of dental age assessment. Hum Biol.

[8] Willems G, Van Olmen A, Spiessens B, Carels C (2001). Dental age estimation in Belgian children: Demirjian's technique revisited. J Forensic Sci.

[9] Nolla CM (1960). The development of the permanent teeth. J Dent Child..

[10] Haavikko K (1974). Tooth formation age estimated on a few selected teeth: a simple method for clinical use. Proc Finn Dent Soc.

[11] AlQahtani SJ, Hector MP, Liversidge HM (2010). Brief communication: the London atlas of human tooth development and eruption. Am J Phys Anthropol.

[12] Kvaal SI, Kolltveit KM, Thomsen IO, Solheim T (1995). Age estimation of adults from dental radiographs. Forensic Sci Int.

[13] Cameriere R, Ferrante L, Cingolani M (2004). Variations in pulp/tooth area ratio as an indicator of age: a preliminary study. J Forensic Sci.

[14] Cameriere R, De Angelis D Fau, Ferrante L, Ferrante L Fau, Scarpino F, Scarpino F Fau, Cingolani M, Cingolani M. Age estimation in children by measurement of open apices in teeth: a European formula. (0937–9827 (Print)). 10.1007/s00414-007-0179-1 17549508

[15] Hegde S, Patodia A, Dixit U (2017). A comparison of the validity of the Demirjian, Willems, Nolla and Häävikko methods in determination of chronological age of 5–15 year-old Indian children. J Forensic Leg Med.

[16] Kumaresan R, Cugati N, Chandrasekaran B, Karthikeyan P (2016). Reliability and validity of five radiographic dental-age estimation methods in a population of Malaysian children. J Investig Clin Dent.

[17] Nik-Hussein NN, Kee KM, Gan P (2011). Validity of Demirjian and Willems methods for dental age estimation for Malaysian children aged 5–15 years old. Forensic Sci Int.

[18] Franco A, de Oliveira MN, Campos Vidigal MT, Blumenberg C, Pinheiro AA, Paranhos LR (2021). Assessment of dental age estimation methods applied to Brazilian children: a systematic review and meta-analysis. Dentomaxillofac Radiol.

[19] Gelbrich B, Carl C, Gelbrich G (2020). Comparison of three methods to estimate dental age in children. Clin Oral Investig.

[20] Wang J, Bai X, Wang M, Zhou Z, Bian X, Qiu C, Li C, Yang Z, Chen G, Ji F et al. Applicability and accuracy of Demirjian and Willems methods in a population of Eastern Chinese subadults. (1872–6283 (Electronic)). 10.1016/j.forsciint.2018.09.006 30286341

[21] Zhou JA-O, Qu D, Fan L, Yuan X, Wu Y, Sui M, Zhao JA-O, Tao JA-OX. Applicability of the London Atlas method in the East China population. LID. 10.1007/s00247-022-05491-8 [doi]. (1432–1998 (Electronic)). 10.1007/s00247-022-05491-8 36066614

[22] Putri AS, Soedarsono N, Nehemia B, Atmadja DS, Ubelaker DH (2021). Age estimation of individuals aged 5–23 years based on dental development of the Indonesian population. Forensic Sci Res.

[23] Subedi N, Parajuli U, Paudel IS, Mallik M (2021). Demirjian's eight teeth method for dental age estimation in Nepalese population. J Nepal Health Res Counc.

[24] Nemsi H, Ben Daya M, Salem NH, Masmoudi F, Bouanene I, Maatouk F, Aissaoui A, Chadly A (2018). Applicability of Willems methods and Demirjian's four teeth method for dental age estimation: cross sectional study on Tunisian sub-adults. Forensic Sci Int.

[25] Brkic H, Galic I, Vodanovic M, Dumancic J, Mehdi F, Anic Milosevic S (2022). The Cameriere, Haavikko, Demirjian, and Willems methods for the assessment of dental age in Croatian children. Int J Legal Med.

[26] Ranasinghe S, Perera J, Taylor JA, Tennakoon A, Pallewatte A, Jayasinghe R (2019). Dental age estimation using radiographs: towards the best method for Sri Lankan children. Forensic Sci Int.

[27] Sezer B, Carikcioglu B, Kargul B (2022). Dental age and tooth development in children with molar-incisor hypomineralization: a case-control study. Arch Oral Biol.

[28] Chaillet N, Nyström M, Kataja M, Demirjian A (2004). Dental maturity curves in Finnish children: Demirjian's method revisited and polynomial functions for age estimation. J Forensic Sci.

[29] Kihara EN, Gichangi P, Liversidge HM, Butt F, Gikenye G (2017). Dental age estimationa in a group of Kenyan children using Willems' method: a radiographic study. Ann Hum Biol.

[30] McCloe D, Marion I, da Fonseca MA, Colvard M, AlQahtani S (2018). Age estimation of Hispanic children using the London Atlas. Forensic Sci Int.

[31] Sousa A, Jacometti V, AlQahtani S, Silva R (2020). Age estimation of Brazilian individuals using the London Atlas. Arch Oral Biol.

[32] Metsaniitty M, Waltimo-Siren J, Ranta H, Fieuws S, Thevissen P (2018). Dental age estimation in Somali children using the Willems et. al model. Int J Legal Med.

[33] Paz Cortes MM, Rojo R, Alia Garcia E, Mourelle Martinez MR (2020). Accuracy assessment of dental age estimation with the Willems, Demirjian and Nolla methods in Spanish children: Comparative cross-sectional study. BMC Pediatr.

[34] Moorrees CF, Fanning EA, Hunt EE (1963). Age variation of formation stages for ten permanent teeth. J Dent Res.

[35] Santana SA, Bethard JD, Moore TL (2017). Accuracy of dental age in Nonadults: a comparison of two methods for age estimation using radiographs of developing teeth. J Forensic Sci.

[36] Yin J, Yang D, Zhang X, Zhang Y, Cai T, Hao Y, Cui S, Chen Y (2020). Diet shift: considering environment, health and food culture. Sci Total Environ.

[37] Zhang L, Ma Y, Yang Z, Jiang S, Liu J, Hettinga KA, Lai J, Zhou P (2019). Geography and ethnicity related variation in the Chinese human milk serum proteome. Food Funct.

