# Supplementary material for: Performance of the London Atlas, Willems, and a new quick method for dental age estimation in Chinese Uyghur children
Source: BMC Oral Health. 2022 Dec 21;22:624. doi: 10.1186/s12903-022-02652-x (PMC9773604; doi:10.1186/s12903-022-02652-x)
Supplement: Supplementary file 1 — Additional file 1. Table S1–S4. Table S1. The intraclass correlation coefficient (ICC) test of the three methods. Table S2. Analysis of ME and MAE between CA and DA using the three methods (years). Table S3. Frequency of dental age estimation accuracy of the three methods in this study by year. Table S4. Age parameter correction for boys and girls. [file 12903_2022_2652_MOESM1_ESM.docx]

Table S1. The intraclass correlation coefficient (ICC) test of the three methods.

|  | London Atlas | Willems method | The quick method |
| --- | --- | --- | --- |
| Intra-examiner agreement | 0.942 | 0.885 | 0.922 |
| inter-examiner agreement | 0.928 | 0.910 | 0.901 |

Table S2. Analysis of ME and MAE between CA and DA using the three methods (years)

| Age | Sex |  | London Atlas | |  |  |  | Willems method | |  | LA-WS |  |  | Quick method | |  | LA-QM |
| --- | --- | --- | --- | --- | --- | --- | --- | --- | --- | --- | --- | --- | --- | --- | --- | --- | --- |
|  |  | ME (SD) | CI | *p*^a^ | MAE (SD) |  | ME (SD) | CI | *p*^a^ | MAE (SD) | *p*^b^ |  | ME (SD) | CI | *p*^a^ | MAE (SD) | *p*^c^ |
| 10 | Boys | 0.09 (0.80) | -0.09, 0.26 | 0.333 | 0.64 (0.48) |  | 1.01 (0.98) | 0.79, 1.22 | < 0.001 | 1.21 (0.70) | <0.001 |  | 0.08 (0.76) | -0.09, 0.25 | 0.345 | 0.62 (0.45) | 0.940 |
|  | Girls | 0.06 (0.93) | -0.14, 0.26 | 0.549 | 0.63 (0.68) |  | 1.02 (1.24) | 0.76, 1.29 | < 0.001 | 1.32 (0.91) | <0.001 |  | -0.20 (0.68) | -0.35, -0.06 | 0.006 | 0.58 (0.40) | 0.007 |
|  | Both | 0.07 (0.87) | -0.06, 0.20 | 0.278 | 0.64 (0.59) |  | 1.02 (1.12) | 0.85, 1.19 | < 0.001 | 1.27 (0.82) | <0.001 |  | -0.07 (0.73) | -0.18, 0.04 | 0.240 | 0.60 (0.42) | 0.030 |
| 11 | Boys | -0.19 (1.21) | -0.51, 0.13 | 0.240 | 0.99 (0.71) |  | 0.69 (1.38) | 0.33, 1.06 | < 0.001 | 1.21 (0.95) | <0.001 |  | 0.09 (0.83) | -0.13, 0.31 | 0.413 | 0.66 (0.50) | 0.031 |
|  | Girls | -0.07 (1.16) | -0.35, 0.22 | 0.641 | 0.91 (0.71) |  | 0.91 (1.23) | 0.61, 1.22 | < 0.001 | 1.25 (0.87) | <0.001 |  | -0.34 (0.79) | -0.53, -0.14 | 0.001 | 0.66 (0.55) | 0.019 |
|  | Both | -0.12 (1.18) | -0.06, 0.20 | 0.278 | 0.95 (0.71) |  | 0.81 (1.30) | 0.85, 1.19 | < 0.001 | 1.23 (0.91) | <0.001 |  | -0.14 (0.83) | -0.18, 0.45 | 0.240 | 0.66 (0.52) | 0.854 |
| 12 | Boys | -0.11 (1.08) | -0.43, 0.21 | 0.491 | 0.89 (0.61) |  | 0.90 (1.40) | 0.48, 1.31 | < 0.001 | 1.29 (1.04) | <0.001 |  | 0.23 (0.68) | 0.03, 0.43 | 0.023 | 0.57 (0.42) | 0.016 |
|  | Girls | -0.09 (1.29) | -0.42, 0.25 | 0.603 | 1.14 (0.60) |  | 0.68 (1.26) | 0.36, 1.01 | < 0.001 | 1.10 (0.92) | <0.001 |  | -0.10 (0.85) | -0.32, 0.12 | 0.389 | 0.66 (0.54) | 0.948 |
|  | Both | -0.10 (1.20) | -0.33, 0.13 | 0.405 | 1.03 (0.61) |  | 0.78 (1.32) | 0.52, 1.03 | < 0.001 | 1.18 (0.97) | <0.001 |  | 0.05 (0.79) | -0.10, 0.20 | 0.533 | 0.62 (0.49) | 0.126 |
| 13 | Boys | 0.01 (1.20) | -0.33, 0.34 | 0.967 | 0.86 (0.82) |  | 0.70 (1.32) | 0.32, 1.07 | < 0.001 | 1.17 (0.92) | <0.001 |  | 0.17 (1.00) | -0.11, 0.46 | 0.221 | 0.85 (0.55) | 0.175 |
|  | Girls | -0.17 (1.21) | -0.44, 0.11 | 0.227 | 0.94 (0.77) |  | 0.80 (1.27) | 0.51, 1.08 | < 0.001 | 1.15 (0.95) | <0.001 |  | 0.04 (0.99) | -0.18, 0.27 | 0.689 | 0.77 (0.61) | 0.036 |
|  | Both | -0.10 (1.20) | -0.31, 0.11 | 0.356 | 0.91 (0.79) |  | 0.76 (1.29) | 0.53, 0.98 | < 0,001 | 1.16 (0.93) | <0.001 |  | 0.10 (0.99) | -0.08, 0.27 | 0.274 | 0.80 (0.58) | 0.013 |
| 14 | Boys | 0.01 (0.81) | -0.23, 0.26 | 0.909 | 0.86 (0.82) |  | 0.51 (1.22) | 0.14, 0.87 | 0.007 | 1.17 (0.92) | 0.004 |  | 0.25 (1.00) | 0.55, 1.66 | 0.104 | 0.85 (0.55) | 0.090 |
|  | Girls | -0.02 (1.33) | -0.37, 0.33 | 0.926 | 0.68 (1.15) |  | 0.68 (1.27) | 0.35, 1.01 | < 0.001 | 1.23 (0.73) | <0.001 |  | 0.03 (0.91) | -0.21, 0.26 | 0.824 | 0.70 (0.57) | 0.767 |
|  | Both | -0.003 (1.13) | -0.22, 0.22 | 0.977 | 0.66 (0.91) |  | 0.60 (1.24) | 0.36, 0.85 | < 0.001 | 1.15 (0.75) | <0.001 |  | 0.12 (0.95) | -0.06, 0.31 | 0.191 | 0.74 (0.60) | 0.211 |
| 15 | Boys | 0.27 (1.12) | -0.11, 0.65 | 0.161 | 0.87 (0.75) |  | 0.30 (1.23) | -0.11, 0.72 | 0.146 | 0.88 (0.90) | 0.790 |  | 0.29 (0.75) | 0.04, 0.55 | 0.025 | 0.58 (0.55) | 0.868 |
|  | Girls | -0.08 (1.13) | -0.38, 0.22 | 0.602 | 0.83 (0.77) |  | 0.53 (1.08) | 0.24, 0.82 | < 0.001 | 0.92 (0.78) | 0.002 |  | 0.30 (0.54) | 0.16, 0.44 | < 0.001 | 0.48 (0.38) | 0.005 |
|  | Both | 0.06 (1.13) | -0.18, 0.29 | 0.635 | 0.84 (0.76) |  | 0.44 (1.14) | 0.21, 0.68 | < 0.001 | 0.90 (0.82) | 0.003 |  | 0.30 (0.63) | 0.17, 0.43 | < 0.001 | 0.52 (0.46) | 0.017 |
| 16 | Boys | 0.61 (1.27) | 0.18, 1.04 | 0.007 | 1.05 (0.92) |  | 1.19 (0.96) | 0.86, 1.51 | < 0.001 | 1.19 (0.96) | 0.003 |  | 0.97 (0.72) | 0.73, 1.22 | < 0.001 | 0.97 (0.72) | 0.039 |
|  | Girls | 0.66 (1.12) | 0.39, 0.92 | < 0.001 | 1.05 (0.76) |  | 1.17 (0.99) | 0.93, 1.40 | < 0.001 | 1.17 (0.99) | 0.002 |  | 0.96 (0.59) | 0.82, 1.10 | < 0.001 | 0.96 (0.59) | 0.018 |
|  | Both | 0.64 (1.17) | 0.42, 0.86 | < 0.001 | 1.05 (0.81) |  | 1.17 (0.98) | 0.99, 1.36 | < 0.001 | 1.17 (0.98) | <0.001 |  | 0.96 (0.64) | 0.84, 1.09 | < 0.001 | 0.96 (0.64) | 0.002 |
| Total | Boys | 0.06 (1.14) | -0.05, 0.18 | 0.241 | 0.83 (0.68) |  | 0.82 (1.22) | 0.65, 0.91 | < 0.001 | 1.16 (0.88) | < 0.001 |  | 0.16 (0.87) | 0.16, 0.34 | < 0.001 | 0.71 (0.55) | < 0.001 |
|  | Girls | 0.05 (1.18) | -0.06, 0.16 | 0.358 | 0.88 (0.80) |  | 0.85 (1.21) | 0.74, 0.96 | < 0.001 | 1.17 (0.89) | < 0.001 |  | 0.09 (0.87) | 0.01, 0.17 | 0.021 | 0.69 (0.54) | 0.343 |
|  | Both | 0.06 (1.13) | -0.02, 0.13 | 0.148 | 0.86 (0.75) |  | 0.44 (1.14) | 0.74, 0.90 | < 0.001 | 1.17 (0.89) | <0.001 |  | 0.30 (0.63) | 0.10, 0.22 | < 0.001 | 0.70 (0.54) | 0.002 |

ME: Mean Error for CA-DA; SD: Standard Deviation; CI: Confidence Interval; MAE: Mean Absolute Error; LA: the London atlas method; WS: the Willems method; QM: the quick method

^a^ The comparison between CA and estimated DA; ^b^ The comparison of the age difference between LA and WS; ^c^ The comparison of the age difference between LA and QM

Table S3. Frequency of dental age estimation accuracy of the three methods in this study by year

| Age |  | London Atlas | |  |  |  | Willems method | |  |  |  | Quick method | |  |
| --- | --- | --- | --- | --- | --- | --- | --- | --- | --- | --- | --- | --- | --- | --- |
|  | MAE<=1 | 1<MAE<=2 | 2< MAE <=3 | MAE > 3 |  | MAE<=1 | 1<MAE<=2 | 2< MAE <=3 | MAE > 3 |  | MAE<=1 | 1<MAE<=2 | 2< MAE <=3 | MAE > 3 |
| 10 | 128 (75.7%) | 37 (21.9%) | 2 (1.2%) | 2 (1.2%) |  | 73 (43.2%) | 64 (37.9%) | 28 (16.6) | 4 (2.4%) |  | 146 (86.4%) | 23 (13.6%) | 0 | 0 |
| 11 | 69 (56.1%) | 42 (34.1%) | 11 (8.9%) | 1 (0.8%) |  | 57 (46.3%) | 45 (36.6%) | 15 (12.2%) | 6 (4.9%) |  | 103 (83.7%) | 14 (11.4%) | 6 (4.9%) | 0 (0%) |
| 12 | 56 (52.3%) | 43 (40.2%) | 8 (7.5%) | 0 (0%) |  | 54 (50.5%) | 36 (33.6%) | 10 (9.3%) | 7 (6.5%) |  | 88 (82.2%) | 16 (15.0%) | 3 (2.8%) | 0 (0%) |
| 13 | 87 (67.4%) | 31 (24.0%) | 8 (6.2%) | 3 (2.3%) |  | 73 (56.6%) | 28 (21.7%) | 24 (18.6%) | 4 (3.1%) |  | 81 (62.8%) | 42 (32.6%) | 6 (4.7%) | 0 (0%) |
| 14 | 83 (80.6%) | 15 (14.6%) | 2 (1.9%) | 3 (2.9%) |  | 46 (44.7%) | 46 (44.7%) | 7 (6.8%) | 4 (3.9%) |  | 78 (75.7%) | 20 (19.4%) | 5 (4.9%) | 0 (0%) |
| 15 | 64 (68.8%) | 24 (25.8%) | 3 (3.2%) | 2 (2.2%) |  | 59 (63.4%) | 28 (30.1%) | 3 (3.2%) | 3 (3.2%) |  | 87 (93.5%) | 5 (5.4%) | 1 (1.1%) | 0 (0%) |
| 16 | 63 (58.9%) | 28 (26.2%) | 14 (13.1%) | 2 (1.9%) |  | 72 (67.3%) | 12 (11.2%) | 17 (15.9%) | 6 (5.6%) |  | 62 (57.9%) | 39 (36.4%) | 6 (5.6%) | 0 (0%) |
| All | 550 (66.2%) | 220 (26.5%) | 48 (5.8%) | 13 (1.6%) |  | 434 (52.2%) | 259 (31.2%) | 104 (12.5%) | 34 (4.1%) |  | 645 (77.6%) | 159 (19.1%) | 26 (3.1%) | 1 (0.1%) |

Table S4. Age parameter correction for boys and girls

| Gender | 10 | 11 | 12 | 13 | 14 | 15 | 16 | Average |
| --- | --- | --- | --- | --- | --- | --- | --- | --- |
| Boys | 16.08 | 16.09 | 16.23 | 16.17 | 16.25 | 16.29 | 16.99 | 16.25 |
| Girls | 15.80 | 15.66 | 15.90 | 16.08 | 16.03 | 16.30 | 16.96 | 16.09 |
